# Perceptions and anticipated factors influencing the adoption of early supported discharge through hospital-at-home

**DOI:** 10.1080/07853890.2025.2537350

**Published:** 2025-07-28

**Authors:** Chong Yau Ong, Ming Jun Kueh, Sungwon Yoon

**Affiliations:** a Department of Transitional Care and Community Medicine, Sengkang General Hospital, Singapore; b Lee Kong Chian School of Medicine, Nanyang Technology University, Singapore; c Duke-NUS Graduate Medical School, Singapore

**Keywords:** Adoption, anticipated factors, early supported discharge, hospital-at-home, patient factors

## Abstract

**Background:**

Hospital-at-home (HaH) is an alternative care model that allows patients to receive quality, individualized care from their homes. This can reduce hospital bed utilization through early supported discharge (ESD) or admission avoidance (AA).

**Objectives:**

This study aimed to explore the patient’s perceptions and anticipated factors of this model of care with a focus on the factors that affect their acceptance of ESD in a multi-ethnic Asian country that remains underexplored.

**Methods:**

A qualitative study was conducted on eligible inpatients with HaH in an acute tertiary hospital in Singapore. Semi-structured interviews were used to explore patients’ views and expectations when deciding to enroll in the HaH. The data were transcribed and analyzed using Braun and Clarke’s thematic analysis framework.

**Results:**

Interview responses were obtained from 24 patients. The key themes identified were: (1) perceived benefits, (2) perceived adequacy of care, (3) potential challenges and barriers, and (4) financial considerations. Perception toward ESD was generally positive with patients citing benefits such as home comfort as well as societal benefits offered by HaH. Potential challenges for patients are diverse, including unsuitable home environments, lack of social support, and technological concerns. The patients’ perceived adequacy of care received from HaH and financial considerations also varied among patients and either positively or negatively affected the willingness for ESD-HaH. The relevance and implications of these factors were further explored, and insights were provided into measures to facilitate the successful implementation of HaH services in Singapore.

**Conclusions:**

This is the first qualitative study conducted on in patients undergoing ESD to explore their anticipated factors influencing the adoption of a relatively new model of care in the Asian community. Addressing the identified themes could improve the adoption of ESD both nationwide and regionally.

## Introduction

The prevalence of non-communicable diseases and rapid population aging is increasing. This has resulted in an inevitable increase in the demand and utilization of healthcare services. This healthcare landscape trend is concerning and is not sustainable. Being among the leading countries with super-aging, Singapore experienced a fair share of acute hospital capacity crunch. Efforts have been made to build more hospital infrastructure, which saw a progressive catch-up post-pandemic. In addition, transitional care facilities and alternative models, such as hospital-at-home, were tapped upon to meet the expansion of overall capacity [1].

Hospital-at-home (HaH) is a viable alternative care model to ease hospital bed shortages [2]. HaH allows patients to receive inpatient care from the comfort of their homes. It can reduce patients’ length of stay (LOS) in hospitals through either early supported discharge (ESD) or complete substitution of ward stay in admission avoidance (AA) [3,4]. ESD in HaH involves helping patients to go home earlier than usual to receive acute or subacute care in their homes for a limited period [5,6]. In the existing literature, HaH has been associated with a higher level of patient satisfaction [7,8], overall reductions in facility cost [2,9], and optimized bed usage in hospital facilities without compromising clinical outcomes [3,4]. While other transitional care services in Singapore primarily focus on post-discharge phone calls and home visits for extended follow-up, the HaH provides a more intensive approach, including inpatient-level care, daily telemonitoring, and intravenous medication administration [10].

Although HaH has been widely accepted in other nations [11], Singapore and most part of Asia are still in early stages of deployment. There were very few Asian works of literature on HaH at the time of writing [8,12–16]. The concept of obtaining acute care outside of a hospital setting has remained unfamiliar. Most existing qualitative studies have been conducted from the viewpoints of healthcare stakeholders and system providers [17]. The acceptability and readiness of patients to adopt this model of care are still underexplored. As patients’ perceptions are at the core of decision-making on utilization of medical services [18], it is crucial to evaluate patients’ views and anticipations of HaH to ensure successful implementation in Singapore.

This study aimed to explore the perspectives of patients on HaH with a focus on the anticipated factors influencing the adoption of ESD when their acute conditions have been stabilized in the initial days at the hospital facility.

## Patients/materials and methods

### Setting and HaH model

In Singapore, the Hospital-at-Home (HaH) program is structured as an extension of inpatient care and is directed by hospital-based physicians, typically family medicine hospitalists or internal medicine specialists. Patients enrolled in HaH are clinically assessed as stable and suitable for early discharge, after which they receive acute-level care at home, including daily medical reviews, nursing visits, and remote monitoring. The care provided is licensed and billed as inpatient-level care under the Ministry of Health’s regulatory framework, ensuring equivalence to hospital-based treatment. The average duration of HaH episodes typically ranges from 3 to 5 days, aligning with the period of subacute care following hospital stabilization.

### Study design

This study used a qualitative study conducted among eligible inpatients in a large public hospital from September to December 2024. In-depth interviews conducted one-on-one physically would allow for delving into the perceptions, anticipations of patients and their acceptance of ESD.

### Participants

The patients were purposively sampled to ensure diversity in age, sex, and enrolment sources in order to capture a broad range of perspectives on ESD. Age groups were stratified to include younger adults, middle-aged adults, as well as older adults with proportions to be representative of the demographics of the program within the institution [19]. Different life stages may influence anticipated factors of care and comfort level with use of technology. Potential gender-related differences in anticipated factors were addressed with balanced representation of the males and females. Recruitment from two enrolment pathways ensure variability in exposure and readiness for HaH, which could influence their anticipations.

Existing inpatients were enrolled through ESD from direct referrals to the HaH program or through a potential patients list. This is an automated list generated daily using use-case diagnosis codes of HaH at the study site. This was used to identify inpatients with common conditions that could be managed in the home setting.

Inclusion criteria included patients admitted to the inpatient wards within the hospital during the study period who had diagnoses manageable in HaH, and whose vital sign were stable (Supplementary Table 1). All emergency department cases/referrals were excluded from this study. Patients who were non-conversant or unable to express their views were excluded from the study. Participants were compensated with vouchers worth Singapore Dollars (SGD) 15 for their time and effort.

### Data collection

One-to-one interviews using a semi-structured interview guide (Supplementary Box S1) were used to explore the patients’ perceptions toward ESD through HaH in the main domains: (1) exposure and understanding of HaH, (2) willingness for ESD, (3) anticipated challenges, and (4) finance bill size and additional incentives. The semi-structured interview guide was developed based on a preliminary review of existing literature on HaH implementation and early supported discharge, alongside insights from pilot service evaluations at the study site. As such, the interview questions were organized around the four pre-specified domains. These domains were selected to reflect likely perceptions and anticipated factors influencing adoption and to ensure comprehensive coverage of issues relevant to patient decision-making. While these domains helped structure the interviews, the subsequent thematic analysis followed the approach of Braun and Clarke. Final themes were not restricted to the pre-specified domains; rather, they were derived from patterns in the data across all interviews. Some correlation between pre-specified domains and emergent themes reflects the alignment between anticipated areas of interest and patients’ actual concerns, but all themes were refined through open coding and iterative discussion among the authors.

Interviews were conducted by two authors (MJK and CYO) who were trained in qualitative interviews. All interviews were conducted before their transition of care through the HaH. Patients were approached by the HaH team and assessed by HaH to confirm the suitability of the program. The interviewer approached the patient to obtain consent for the interview. The interviews were conducted at the bedside of the cubicle or room. The environment was assessed as quiet and conducive before the interview. The audio recordings were transcribed verbatim and de-identified. Brief field notes were made adjunct to the audio recordings. For two Mandarin-speaking patients, interviews were conducted in Mandarin, transcribed, and translated into English by an interviewer who was proficient in both languages. Recordings were proofread (MJK, CYO) before being computed in Excel sheets. The rapid qualitative interviews lasted between 10 and 42 min. No repeated interviews were conducted. Transcripts were not returned to participants for comments owing to manpower constraints in the lean study team.

### Data analysis

Thematic analysis was performed using Braun and Clarke’s six-phase thematic data analysis method [20]. The six phases are briefly outlined as follows: (1) Familiarization with the data through reading and re-reading, (2) systematic data coding, (3) generating initial themes from codes, (4) reviewing themes for consistency, (5) refining the theme, and (6) reporting writing. No a priori conceptual framework is employed to derive the initial code sets. Two authors coded the data (MJK and CYO) with another author (SY) adjudicating and discussing the inputs whenever there were differences. The data were then computed into NVivo 14 (Lumivero, DO) and coded using both deductive and inductive techniques. The inductive approach allows themes to emerge through the identification of the core meanings of the data relevant to the objectives of the study [21]. The deductive approach was used to test, refine, or refute existing hypotheses or assumptions. This process was completed until thematic saturation was reached [22]. Findings were reported according to the Consolidated Criteria for Reporting Qualitative Studies (COREQ) checklist [23].

#### Ethical approval

The study was determined by the Centralized Institutional Review Board (CIRB) as not requiring an institutional review board (IRB) as it delves into the views and perspectives of patients on this model of care, especially the barriers and facilitators (CIRB Ref: 2024/2293). Under the IRB, surveys, interviews, and observations of public behavior can be exempt if conducted to protect participant anonymity; essentially, the research poses minimal risk of harm to participants and does not involve identifiable information. Eligible patients were approached and invited to participate in this study. Participation in the study was voluntary and written consent to publish the quoted statements from participants was obtained. This study adheres to the Declaration of Helsinki regarding research involving human subjects.

#### Author reflexivity statement

The study team comprised an academician (SY), clinician (CYO), and undergraduate medical student (MJK). SY has extensive expertise in qualitative research methodologies and public health and health service research. CYO is a committee in the National Mobile Inpatient Care @Home Singapore workgroup and is heavily involved in the set-up of hospital-at-home in the study site. MJK is an undergraduate in the medical program with research interests and has been trained in basic qualitative research. He received an attachment at the study site to understand the program before conducting the study. Our study was informed by a knowledge gap regarding perception and acceptability. Through this research, the authors hope to use the findings to inform health policy modifications and improvements. All authors are based in Singapore and understand the cultural norms and preferences of Singapore’s multi-ethnic society. There was no relationship with participants who were established before the study commenced. The participants were blinded to the interviewer and investigators.

## Results

A total of 35 patients were approached as potential patients for ESD, with 11 patients declining because of reasons such as such as wanting to rest, not comfortable in sharing their thoughts, and only able to speak in native languages. This resulted in a final sample size of 24 patients. The mean age of the interviewed patients was 46.9 (SD ± 20.9). 54.2 Of the patients, 54.2% were male. The patients were of multiple ethnicities (Table 1). The most common primary diagnoses are gastroenteritis and cellulitis. All patients had no prior experience with ESD or HaH.

**Table 1.** Patient descriptive characteristics of interviewed patients (*n* = 24).

Characteristics	Patients (*n* = 24)
Age (Mean ± SD)	46.9 ± 20.9
Age distribution (%, *n*)	
<30	20.8 (5)
30–60	41.7 (10)
>60	37.5 (9)
Gender (%, *n*)	
Male	50.0 (12)
Female	50.0 (12)
Race/ ethnicity (%, *n*)	
Chinese	50.0 (12)
Malay	33.3 (8)
Indian	12.5 (3)
Others	4.2 (1)
Primary diagnosis (%, *n*)	
Gastroenteritis	25.0 (6)
Cellulitis	16.7 (4)
Dengue	12.5 (3)
Urinary tract infection	8.3 (2)
Pyelonephritis	4.2 (1)
Pneumonia	4.2 (1)
Diabetes Mellitus	4.2 (1)
Fluid overload	4.2 (1)
Rhabdomyolysis	4.2 (1)
Migraine	4.2 (1)
Gout flare	4.2 (1)
Atypical chest pain	4.2 (1)
Fever in returning traveller	4.2 (1)
Length of stay, LOS (Mean ± SD)	3.18 ± 2.34

**Figure 1.**
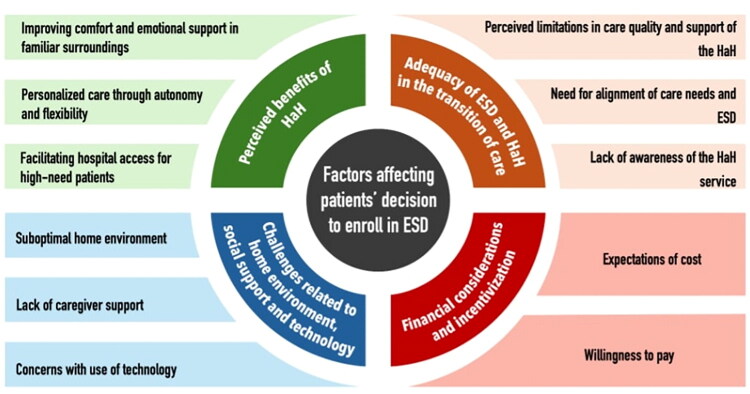
Factors affecting patient’s decision to enroll in ESD.

Four themes (perceived benefits of HaH, adequacy of ESD and HaH in the transition of care; challenges related to home environment, social support, and technology; and financial considerations and incentivization) emerged from the analysis (Figure 1). The themes were also interrelated, suggesting the complexity of decision-making for ESD beyond the categorization of anticipated factors.

### Theme 1 – Perceived benefits of HaH

This theme highlights the perceived benefits of HaH. These are the main drivers that would increase patients’ inclination toward adopting the HaH model of care. This theme can be divided into the following three subthemes**.**

### Improving comfort and emotional support in familiar surroundings

Patients highlighted the comfort and emotional ease associated with receiving care at home compared with staying in the hospital. They described the challenges in adapting to an unfamiliar hospital environment, particularly regarding sleep and privacy. This suggests that uninterrupted rest was a significant benefit to the home environment, enhancing recovery experiences.

I mean, if I go back home, rather than stay here, I have uninterrupted rest. Sometimes you have, suddenly, nurses coming in, trying to resuscitate, disturbing your rest. (Patient 11, 35-year-old)

Many researchers have emphasized the importance of having a nearby family during recovery. Being at home allowed patients to stay connected with their loved ones, receive emotional support, and reduce feelings of isolation.

Yes, I think another big thing is that my family will always be around me …. It is comforting to know that my family members will be around me if I need anything. (Patient 2, 22-year-old)

Personalized care through autonomy and flexibility. Participants perceived the HaH model as a means of improving flexibility and personalization of care in a home-based environment. This was especially apparent for some patients who expressed frustration with the restrictions of the hospital setting. They shared a strong preference for managing their health needs and daily routines. Additionally, patients valued the ability to perform a wider range of activities, which allowed them to balance their personal, social, and professional responsibilities during recovery and fulfill other commitments they may have.

…I can monitor [my condition] on my own rather than having someone to fuss over me all the time…. I’d rather food tailored according to what I need…. (Patient 10, 31-year-old)

I can walk around my house, sit around my house or on the TV or anything. My friends can just come in and visit me. (Patient 7, 68-year-old)

### Facilitating hospital access for high-need patients

Patients felt that ESD to HaH would address hospital resource constraints, particularly bed shortages. Many expressed interests in transitioning to home-based care and recognized the opportunity it creates for critically ill patients to access hospital beds in a timely manner.

Because for me I feel better now compared to yesterday…. Honestly, I just feel like I’m just wasting someone’s space lah……. So, I was thinking if let us say I will take up this program, then the ward can be given to them faster. (Patient 18, 24-year-old)

This indicates that some patients showed an altruistic understanding of the potential benefits of the HaH model for the broader healthcare system.

### Theme 2 –Adequacy of ESD and HaH in the transition of care

Patients’ willingness to adopt HaH care was also affected by their views toward the adequacy of ESD and HaH care services. These perceptions were shaped primarily by concerns that the quality of care in HaH might be limited relative to inpatient care**.** Participants with higher perceived care needs, coupled with a lack of awareness of the service, also highlighted safety concerns about being treated and nursed outside actual hospital facilities.

### Perceived limitations in care quality and support of the HaH

Patients anticipated that the quality of care received from HaH may be limited compared to ward stay. For example, one patient contrasted the convenience of hospital care, where pressing a button summons immediate help, with the isolation of home-based care under the HaH. This finding underscores the perceived gap in the immediacy of medical assistance at home. The lack of direct access to doctors and medical equipment was also a recurring theme, reflecting concerns that remote monitoring and virtual care may not be sufficient to address all medical needs.

Over here, I can just press the button then the service comes straight to me. But over there, at home, I will be alone. (Patient 12, 44-year-old)

…we don’t have the doctors on site. So, there’s only so much you can observe through Zoom and all. (Patient 10, 33-year-old)

Need for alignment of care needs and ESD Patients who thought that their condition was well controlled were more comfortable with HaH, as they believed their lower care needs could be effectively managed in a home care setting. However, patients commented that if they still experienced poorly controlled symptoms, had difficulty performing daily activities, or questioned the stability of their condition, they would be uncertain if ESD would adequately meet their care needs.

I also get to receive some care, maybe not at the same level but still good enough. And maybe because I am starting to feel better, so I would not need the same amount of care that I am receiving right now. (Patient 24, 24-year-old)

I think hospitalized care is the best during the first few days. [I was] unable to get up at all because of the pain, the fever, and the giddiness. Overall, taking care of [my] needs…. (Patient 23, 31-year-old)

It’s more concern of immediate attention. What if the temperature should be increased and I got 40-degree temperature again… I got to wait for attention again…. So, at home, it’s a risky setting. (Patient 12, 44-year-old)

The mismatch between their higher perceived care needs and the lower levels of care expected from ESD resulted in safety concerns and more reservations toward ESD to HaH.

### Lack of awareness of the HaH service

Only 3 of the 24 patients had heard of the HaH model of care before referral or interview. This resulted in greater uncertainty regarding the care they received. Several patients noted that having more information earlier could positively influence their decision-making processes.

Maybe it’ll help if I know about the program earlier…. I have not heard about it before. Yeah, so I can make a sort of have a discussion with my family at the start of my admission. (Patient 24, 24-year-old)

I know people say every time you will get the same service as you are getting in the hospital. However, I think another concern is how much is that true. I think people are a bit afraid to find out because they are not sure if they would get the same service they are getting at home as well. (Patient 9, 28-year-old)

Lack of experience with HaH or knowledge of its safety and effectiveness created more doubts among the patients who already had reservations about the adequacy of care received with ESD.

### Theme 3 – Challenges related to home environment, social support, and technology

This theme explores the potential barriers and challenges patients face when considering enrollment in HaH. These barriers are diverse and depend heavily on the patient’s sociodemographic and environmental conditions. Three key themes have emerged.

### Suboptimal home environment

Patients were more likely to turn down ESD when they felt that their home environment was unsuitable. Some patients expressed concerns that disruptions at home would hinder their rest and recovery. Other patients were more concerned with physical constraints such as space to accommodate drip stands, clinical teams, and even the presence of co-living persons who were not family members. This makes HaH delivery inconvenient and impractical.

I’m not willing to go because at home may not be the best setting. I lost my sleep when I had it. So, in the hospital, [I can] just sleep. At home, I will not be able to sleep, I will just be with my children and playing [with them]. So, it’s me time here now. (Patient 12, 44-year-old)

But those who are renting out, I think that might be the concern and worry because if it’s just in a room, it’s quite difficult to have all the facilities and like to always go out and come in with the drips and all. (Patient 9, 27-year-old)

These concerns highlight how unsuitable home environments may act as significant barriers for patients to enroll in a HaH.

### Lack of social support

The absence of a caregiver at home is frequently cited as a major barrier, with many patients feeling unsure about managing their care independently. Caregivers can support patients by assisting them with their care needs and acting as a safeguard against the possible deterioration of their condition.

I know my wife can help me at home. But if let’s say, I’m a widow or I’m alone, then I would not want to go home… (Patient 5, 74-year-old)

If there was someone, not only parents there was someone to look after you, that would be a factor that they would not mind going back to. I would still take it on. Yeah, if there was someone, my parents were there, I would still take it on. (Patient 9, 28-year-old)

### Concerns with the use of technology

Most patients felt comfortable using technology to receive remote care, citing prior experience with similar digital platforms. In contrast, some concerns about technology proficiency have been raised by older patients.

My generation is less tech-savvy. So, even the choice I would still prefer, you know, face-to-face. (Patient 5, 74-year-old)

That is a problem because not everybody is computer savvy. For me, when I worked as a mechanic, I did not touch my computer. My laptop is my only software after my equipment. If you ask me to write an email, send a text, or something else I may have difficulties. (Patient 7, 68-year-old)

Beyond technological comfort, some patients are worried about the effectiveness of teleconsultations in providing quality medical care. Patients were concerned about their ability to convey their symptoms clearly through teleconsultations and the lack of thorough physical examinations, which led some patients to prefer in-person consultations.

[If] at home on Friday, a new bruise appears… I cannot explain like how it feels like, they need to physically touch. (Patient 3, 33-year-old)

… I talk very slow, very difficult [to talk properly]. So, talking to people through the phone, a bit difficult. (Patient 15, 81-year-old)

### Theme 4 – Financial considerations and incentivization

Patients’ views on cost include expectations of cost, and their willingness to pay for HaH constitutes the subthemes under financial considerations and incentives.

### Expectations of cost

When patients were asked how much they would expect to pay for HaH, most patients commented that they expected the cost to be lower than their ward stay. As seen in Table 2, this was because they felt that they were using fewer hospital facilities and receiving a lower level of care.

**Table 2.** Themes, and corresponding subthemes and quotes.

Theme 1 – Perceived benefits of HaH				
This theme highlights the perceived benefits from HaH. These form the main drivers that would increase patients’ inclination toward adopting this model of care.				
Subtheme	Description of subtheme	Subcodes	Description of subcodes	Supporting quotes
Improving comfort and emotional support in familiar surroundings	Patients felt that receiving treatment from home in HaH would be more conducive more their rest and recovery.	Comfort and familiarity	Patients feel that they would be more comfortable resting at home as they do not have to adjust to a new environment.	‘I guess as somebody who don’t really get used to sleeping in the new place. So like last night (in the ward), totally can’t sleep at all…’ (Patient 4, 58-year-old) ‘For comfort-wise, I would say at home would be better, because it’s my own place right. Then can watch TV and do my daily routine. I can watch TV, I can be around my kids, my family members’. (Patient 23, 38-year-old)
		Privacy	Patient also felt that receiving treatment at home offered more privacy which was beneficial to perceived comfort as well as avoidance of disruptions.	‘Okay, I mean if I’m at home I feel much more comfortable you know. Like let’s say you over here… because [I’m] sharing with people, I won’t say awkward but, you know I feel like walking around but I [feel embarrassed] to walk around’. (Patient 18, 24-year-old) ‘I mean, if I go back home, rather than stay here, I have uninterrupted rest. Sometimes you have, suddenly, nurses coming in, trying to resuscitate, disturb your rest’. (Patient 11, 35-year-old)
		Prescence of family	The presence of the patient’s family can offer emotional support which allows the patient to feel more at ease. This is less readily available in the hospital.	‘Yes, I think another big thing is that my family will always be around me…. it is comforting to know that my family members will be around me if I need anything’. (Patient 2, 22-year-old) ‘Being at home, firstly, it’s my family members. So, I think having them around is a good thing because I won’t feel socially isolated from people’. (Patient 24, 25-year-old)
Personalized care through autonomy and flexibility	Patient felt that an ESD allows patient to have more control over their own environments and care. They can better adjust their needs due to greater access to home resources and the lack of restrictions associated with hospital stay	Autonomy over care	In the hospital, patients are required to follow strict rules and routines with little control over things like lighting of their environment as well as their meals. Although they still have to follow certain instructions from the medical team, patients felt HaH arrangements will allow for more control over their care.	‘So I guess it’s something that I can just monitor [my condition] on my own rather than having someone to fuss over me all the time…. I rather food tailored according to what I need. [sometimes] I really got no appetite and just wanted soup. [But] they would give me a whole set then I cannot finish them. So, I feel very sad that I’m throwing food away’. (Patient 10, 31-year-old) ‘Home will be in terms of sound and lighting. I mean, I can draw the curtains, the noise level I can control. But here in what environment, I have to appreciate that others need the lighting. And then the sound is unavoidable in the sense that there will be other patients’. (Patient 5, 74-year-old)
		Flexibility in activities	Patients felt limited by the things they were able to do in the hospital due to space restrictions and lack of access to their belongings. This often resulted in lower levels of satisfaction and boredom amongst patients. They felt incentivized to go home as they would be able to do more to pass the time as they continue to recovery.	‘I have to stay here. Then I want to move out, I also cannot move out. So, at home, I can walk around my house, sit around my house, watch the TV or anything. My friends can just come in and visit me. But in hospital, you cannot [have] 4 or 5 person [visiting] you’. (Patient 7, 68-year-old) ‘I think they would want to be more free, like just to be at home, rather than being very restricted in the hospital. Especially when you have your own space, you can easily walk around, you can do things, whatever that you like. Here, you are just in a room, and you cannot do much. So, I think that is one, people, they would like that in the comfort of your own home. (Patient 9, 28-year-old)
		Fulfill other commitments	Receiving treatment from home was also an avenue for patients to fulfil commitment they would otherwise not be able to fulfil in the hospital. Examples of this include taking their of children and working from home.	‘I do have the ability to work from home, so I can just do that this week and then yeah…. because I can’t really be on calls all day long if I’m just here’. (Patient 17, 22-year-old) ‘Yeah, like today my purpose is I want to care for my child. So, if next time maybe if I have a reasonable or more priority to get a care at home’. (Patient 8, 37-year-old)
Subtheme	Description of subtheme	Subcodes	Description of subcodes	Supporting quotes
Facilitating hospital access for high-need patients	Patients felt that ESD to HaH would ease the issue of bed shortages.	Ease bed shortage	Patients pointed that overcrowding in acute hospitals is a known issue and ESD would be an effective solution to this problem.	‘I also appreciate the crowding in hospitals and waiting for beds and all that lah. [I should] give others who need it more than me to have the hospital well lah’. (Patient 5, 58-year-old) ‘I think the number of beds has been a known issue. You guys coming up with this particular program that is actually, it’s good lah, I feel it’s good’. (Patient 11)
		Fair allocation of resources	Patients feel that in cases where their condition no longer warrants hospital ward stay, it would be fairer to give up their space in the hospital so people which worse medical conditions can receive the level of care they require.	‘Like opening a space like the hospital because for more like more needed patient. Because for me I feel better now compared to yesterday…. And honestly, I just feel like I’m just wasting someone’s space lah……. So, I was thinking like if let’s say I’ll take up this program, then the ward will be given to them faster’. (Patient 18, 24-year-old) ‘But with this assisted program, someone we don’t know down there at the ED, someone could be needing this bed more than us. So, I think that it’s okay to give up the bed’. (Patient 3, 33-year-old)
Theme 2 – Adequacy of ESD and HaH in the transition of care				
Decision making was also affected by patients’ views had toward the adequacy of care provided by HaH. These perceptions are affected by the expectation of the level of care in HaH relative to their perceived care needs				
Subtheme	Description of subtheme	Subcodes	Description of subcodes	Supporting quotes
Perceived limitations in care quality and support of the HaH	Patients believed that the level of care received from HaH will be lower compared to ward stay.	Limited capabilities of monitoring	Limitations associated with receiving care at a remote site due to the absence of medical professionals and equipment on site	‘…we don’t really have the doctors on site. So, there’s only so much you can observe through zoom and all’. (patient 10, 33-year -old)
		Lower quality of medical attention	Patient felt monitoring will be less frequent compared to the hospital and would have lower level of care during HaH.	‘[there is] uncertainty because if you stay at a hospital and you receive care like 24/7 and you take your medications on time then you’re probably gonna make a full recovery but at home [there is] a slight a bit of uncertainty’. (Patient 13, 26-year-old)
		Less immediate medical attention	Speed medical attention accessible to them with HaH would be inferior to staying in the ward.	‘Over here, I can just press button then the service come straight to me. But over there, at home I will be alone’. (Patient 12, 44-year-old)
Need for alignment of care needs and ESD	Patients with different control of symptoms and medical conditions had different options whether their care needs could be adequately met by HaH.	Lower care needs	Patients who thought that their condition is well controlled were more comfortable with HaH as they believe they have lower care needs that can be met by home arrangements.	‘I also get to receive some care, maybe not at the same level but still good enough. And maybe because I’m starting to feel better, so I wouldn’t need the same amount of care that I’m receiving right now’. (Patient 24, 24-year-old) ‘Well, I feel like I don’t need like 24/7 care. I feel like I can take care of myself and also, I would rather just be home’. (Patient 17, 22-year-old)
		Higher care needs	Patients commented that if they still experienced poorly controlled symptoms, had difficulty in carrying out daily activities or questioned the stability of their condition, they would be uncertain if ESD would adequately meet their care needs.	‘I think hospitalized care is the best during the first few days. [I was] unable to get up at all because of the pain, the fever and the giddiness. Overall, taking care of the [my] needs…’. (Patient 23, 31-year-old) ‘It’s more concern of immediate attention. What if the temperature should be up and I got 40-degree temperature again… I got to wait for attention again…. So, at home, it’s a risky setting’. (Patient 12, 44-year-old)
Subtheme	Description of subtheme	Subcodes	Description of subcodes	Supporting quotes
Lack of awareness of the HaH service	As most patient were unfamiliar with HaH, there was more uncertainty regarding the care they would receive.	Have not previously heard of HaH	Most patient have not heard of ESD or HaH prior to the interview or referral to the service.	‘I know people every time say you will get the same service as you are getting in hospital. But I think another concern would have been like how much is that really true? I think people are actually a bit afraid to find out because they are not sure if they would actually get the exact same service they are getting at home as well’. (Patient 9, 28-year-old)
		More information regarding HaH would be useful	Several patients noted that having more information earlier could have positively influenced their decision-making process.	‘Maybe it’ll help if I know about the program earlier…. I have not heard about it before. Yeah, so I can make a sort of have a discussion with my family at the start of my admission’ (Patient 24, 24-year-old) ‘a brochure to introduce the program. That would be useful as well. And then that would be something that I can refer to…’ (Patient 10, 31-year-old)
Theme 3 – Challenges related to home environment, social support and technology				
This theme explores the potential barriers and challenges patients face when considering enrollment in HaH. These barriers are diverse and depend heavily on patients’ sociodemographic and environmental conditions.				
Subtheme	Description of subtheme	Subcodes	Description of subcodes	Supporting quotes
Suboptimal home environment	Patients were more likely to turn down ESD in cases where they felt their home environments were unsuitable.	Disruptive home environment	Some patients were concerned that disruptions present at home would hinder their rest and recovery.	‘I’m not willing to go because at home may not be the best setting. I lost sleep when I had this. So, in hospital [I can] just sleep. At home, I will not be able to sleep, I will just be with my children and playing [with them]. So it’s me time here now’. (Patient 12, 44-year-old) ‘When it comes to recovery process, it shouldn’t be a chaotic condition at home…. On top of that, some of us may not have… you know those low incomes, they have to share a lot of things at home. So, again, they may not have access to probably the Wi-Fi or anything. Probably the house is too cluttered. For this I might have to say, I would rather stay here lah’ (Patient 11, 35-year-old)
		Constraints of home environment	Other patients were more concerned with physical constraints such as space that would make HaH inconvenient and impractical. This is especially applicable in patients who are renting out places for their accommodation.	‘But those who are renting out, I think that might be the concern and worry because if it’s just in a room, it’s quite difficult to have all the facilities and like to always go out and come in with the drips and all’. (Patient 9, 27-year-old)
Lack of caregiver support	The absence of a social support at home was frequently cited as major barrier with many patients feeling unsure about managing their care independently.	Family/Caregivers	Caregivers can support patient by assisting them in their care needs and act as a safeguard in the possible situation of their condition deteriorating.	‘I know my wife can help me at home. But if let’s say I’m a widow or I’m alone, then I would not want to go home…*’* (Patient 5, 74-year-old) ‘If there was someone, not only parents, if there was someone to look after you, that would definitely be a factor that they would not mind going back. I would still take it on. Yeah, if there was someone, my parents were there, I would still take it on’. (Patient 9, 28-year-old)
		Other forms of social support	In absence of caregivers, patients with robust social support systems with their friends also feel more confident in enrollment in HaH.	‘Even if I’m alone at home, I have got a good support system. But even if I’m alone, I have my friends who won’t make me feel alone’. (Patient 10, 31-year-old) ‘True… Well, not really, because I do have friends anyway. I feel like nowadays we can like stay in contact pretty easily, so if I ever need someone, I know that someone will be here for me. And also my neighbors, they’re quite nice, so if I ever need anything, I know they’ll be here’. (Patient 17, 22-year-old)
Subtheme	Description of subtheme	Subcodes	Description of subcodes	Supporting quotes
Concerns with use of technology	Although most patients felt comfortable using technology to receive remote care citing prior experiences with similar digital platforms. In contrast, some concerns about proficiency in technology were raised by older patients.	Comfortable with use of technology	Majority of patient had no issues with the use of technology as they have experience which such technological mediums (e.g. video calls, tele consults).	‘I tried [teleconsultations] before with polyclinic. I think it’s good enough’. (Patient 8, 37-year-old) ‘No, I feel like because of my age also, I’m really used to it, even my work, I mean I work from home a lot, so I’m used to doing meetings online, I’m used to using like online tools, maybe for some like elderly people it might be harder to use, but it’s pretty common in my generation I feel like’. (Patient 17, 22-year-old)
		Concerned on proficiency	In particularly the older adults, some reported concerns about their ability to use technology which would make enrollment in the problem difficult.	‘My generation is less tech-savvy. So, even the choice I would still prefer, you know, face-to-face’. (Patient 5, 74-year-old) ‘That is a problem because not everybody is a computer savvy. For me, when I was working as a mechanic, I didn’t touch my computer. My laptop is only my software after my equipment. If you ask me to write an email, send a text or something else I may have difficulties’. (Patient 7, 68-year-old)
		Concerns of limitations of technology	Some patients reported that use of technology in HaH may still come with some limitations including reduce effectiveness of communication and inability to conduct thorough of clinical assessments.	‘[If] at home on Friday, a new bruise appears. So if I show them, I cannot explain like how it feels like, they need to physically touch’. (Patient 3, 33-year-old) ‘Because when it comes to talking, [my talking is] not very good… I talk very slow, very difficult [to talk properly]. So, talk to people through the phone, a bit difficult’. (Patient 15, 81-year-old)
Theme 4 – Financial considerations and incentivization				
Patients views on cost includes the expectations of cost and their willingness to pay for HaH				
Subtheme		Subcodes		Supporting Quotes
Expectations of cost	Most patients commented that they expected the cost to be lower compared to ward stay.	Reduced medical attention	Patients feel that as there will be less medical attention given to them during HaH, the costs associated with HaH services should be reduced.	‘Overall, because it uses my facilities at home and I do not have a lot of attention. So, it should be a lot lower than [staying in the hospital]’ (Patient 12, 44-year-old) ‘If I’m at home doing everything myself, I might as well stay in the hospital and get everything done there’. (Patient 6, 63-year-old) _
		Lack of use of hospital facilities	Patients feel that as they are not using physical facilities of the hospital such as the aircon, hospital bed and food, the cost that they are charged should be reduced	‘I’m not occupying a space in the hospital. Then it should be like much cheaper’. (Patient 18, 24-year-old) ‘I thought that because you don’t have to serve meals, you don’t have to use the thermometer, we self-help, you should be having more significant savings’. (Patient 19, 67-year-old)
Willingness to pay	There is a diversity of opinions on the willingness to pay for HaH.	Incentive of cost reductions	Some patient felt that a cost reduction would be a helpful incentive for enrollment in ESD to HaH.	‘I think it would be a lot more appealing’. (Patient 10, 33-year-old) ‘I think it would definitely make people to think twice, and they would be a bit more keen, rather than if it is going to be around the same price’. (Patient 9, 28-year-old)
		Willingness to pay same cost	Some patients would still be willing to pay for HaH service if the price is the same as ward stay. For some patients this was because it was able to make use of subsidies and insurance schemes that made the service affordable to them; while other patients believed that the benefits of HaH were sufficient to justify the costs.	‘Because it’s subsidized and it can be used [with] Medisave, then it’s okay. But if it’s private to pay in cash, that would be quite expensive to do’. (Patient 23, 38-year-old) ‘Like if I had to choose for around the same cost, even if it was more expensive, I would still choose to be at home. But then again, I also have like international insurance, so I know that I will get my money back. So, I would rather just choose something that’s more comfortable for me’. (Patient 17, 22-year-old) ‘If you say the cost typically will be much lower, then I would think that, yes, why not? Maybe a 30% cost difference? Yes, I would say that that makes an attraction… The expenditure of the medical is very expensive’ (Patient 2, 55-year-old)

I’m not occupying a space in the hospital. Then it should be much cheaper. (Patient 18, 24-year-old)

I thought that because you don’t have to serve meals, you don’t have to use the thermometer, we self-help, you should be having more significant savings. (Patient 19, 67-year-old)

### Willingness to pay

There is diversity in the willingness to pay for HaH. Some patients believed that cost reductions would incentivize them to enroll in ESD, while others mentioned that they would still be willing to pay for HaH services if the price was the same as the ward stay. They attributed this to the reasonability and affordability of HaH, as they were still able to use subsidies and insurance schemes. Some patients believed that the benefits of HaH were sufficient to justify the cost, with some willing to pay more for the service.

Because it’s subsidized and it can be used [with] Medisave, then it’s okay. But if it’s private to pay in cash, that would be quite expensive to do. (Patient 23, 38-year-old)

Like if I had to choose for around the same cost, even if it was more expensive, I would still choose to be at home. But then again, I also have international **insurance**, so I know that I will get my money back. So, I would rather choose something more comfortable for me. (Patient 17, 22-year-old)

## Discussion

This study explored the perceptions and anticipated factors that influence patients’ decisions to adopt ESD. The attractiveness of ESD to HaH includes improving comfort and emotional support in familiar surroundings, personalized care through autonomy and flexibility, and facilitating hospital access for patients with high needs. The main barriers identified included suboptimal home environments, lack of social support, and concerns regarding technology. Additionally, patients should consider the adequacy of HaH to meet their transitional care needs and the cost of HaH when making their decision for ESD.

Patients generally had a positive outlook toward ESD, and most were willing to consider it. Consistent with both local and international studies, our findings suggest that home environments offer greater comfort and flexibility, enhancing rest and overall patient satisfaction [24–26]. Our study also highlights the importance of home environment conduciveness, where constraints of space or disruptive home environments could act as potential barriers for ESD to HaH [27]. Traditional housing is not designed to accommodate healthcare equipment, healthcare providers, or remote care infrastructure; this is heightened in Singapore, where over 80% of residents live in high-rise public housing flats provided by the Housing and Development Board on leasehold land [28].

Our findings also suggest that the perceived adequacy of ESL and HaH care is a key factor for patients. Similarly, previous studies have shown that the feeling of safety and confidence in HaH services is a key determinant for enrollment in the program [29–31]. The need for escalation and re-admission back to the actual hospital facility in case the patient deteriorates or undergoes a change is a pragmatic concern with this model of care [32]. The lack of awareness of HaH is especially evident in our study, likely because most patients lacked exposure to this model of care, resulting in apprehension toward enrolling in HaH [31,33,34]. This is despite nationwide communication through media such as television and newspapers during the pilot phase in 2023 and the mainstreaming phase in 2024. Similar challenges have been reported in UK and Australia, where successful HaH programs were supported by sustained public information campaigns that included information brochures in high volume patient fronting areas such as the emergency departments, GP clinics, and pharmacies; testimonials on the hospital websites and social medias; and targeted outreach through community health workers [17,25]. Adopting these strategies in Singapore by integrating HaH in healthcare portals, broadcasting multilingual education videos, and collaborating with primary care networks can improve public familiarity and normalize HaH as one of the standard care option. Good communication between the medical team and patients, as well as a thorough clinical assessment before transfer to HaH, will be key pillars in alleviating safety concerns while building confidence in HaH services [27]. The desire to help alleviate hospital overcrowding was a unique finding in our study, reflecting the potential for public health campaigns to frame HaH as not only beneficial to individuals but also to the broader healthcare system.

Concerns about technological proficiency, particularly in adopting telemedicine, have been widely documented [35–37]. Willingness to adopt technologies is found to be lower among older seniors than younger seniors [38]. Addressing these challenges will require targeted interventions, such as digital literacy programs for older adults and the use of hybrid care models that combine remote and in-person visits for more complex cases [39–41]. At present, most remote patient monitoring requires minimal diligence from the patient’s end to submit vital signs at prespecified time intervals. This can be challenging for older people who are not technology savvy. Even though they were taught how to use the monitoring devices and equipment before going home, some patients encountered difficulties in timely submissions. Older persons are less intuitive in navigating more complex interfaces, digital devices, and products that they do not own in comparison to young adults [37,42]. Additionally, integrating more user-friendly and advanced monitoring systems may help address patients’ technological concerns [43].

The importance of caregivers in the successful provision of HaH was emphasized in our study [8,27,31]. Although our study did not include the input of caregivers in the decision-making, we found that they still are a key consideration for patients. Caregivers play a significant role in providing physical and emotional support for patients and act as strong enablers to enroll in HaH [24]. In Singapore, the culture of strong family involvement in medical decision-making further solidifies the need to involve caregivers in the discussion of decisions regarding HaH [43].

The healthcare financing model in Singapore is a mixed financing system, in addition to direct government subsidies for hospital admissions and polyclinics, Singapore relies on the ‘3M’ system of programs- Medisave, MediShield, and Medifund. Any costs above the claim limit incur out-of-pocket payments to encourage responsibility and discourage overconsumption. One of the out-of-pocket costs includes the COVID-19 Virtual Ward, which was a financial disincentive leading to some patients choosing to remain hospitalized in the actual hospital facility to utilize standard healthcare financing [16]. Regarding matters of cost, keeping the service affordable to patients is important in maintaining the attractiveness of HaH. We found that the majority of patients would only consider HaH if its cost was the same as or lower than the ward stay [8,27]. However, there are other indirect costs to be considered beyond bill size for the patient and family members, such as time taken off from work, and time, and transportation costs to incur if the patient is to be visited in the actual hospital facility [19]. Existing studies examining the cost of HaH versus inpatient hospitalization found cost differences in favor of HaH; however, many of these were overestimated [44]. Future research should focus on the cost-effectiveness of HaH to ensure cost savings for both patients and healthcare institutions.

While this study was conducted in a public hospital setting in Singapore, several findings have potential for applicability in other densely populated urban settings. The impact of housing constraint such as limited space, co-living arrangements, and lack of private area may similarly challenge the uptake of HaH in other urban cities with high-rise housing, such as Hong Kong, Seoul, or New York. Moreover, Singapore’s high digital literacy combined with strong familial involvement in healthcare decision-making offers lessons for implementing HaH in cultures that are tech-ready but socially interdependent. These insights highlight the need to adapt HaH models not only to clinical eligibility but also to consider the social and environmental contexts, for successful adoption.

This study had a few limitations. First, the interviews were conducted before the transfer to HaH, therefore patients’ expectations may not accurately reflect their actual experiences with HaH. Further interviews with patients after discharge from HaH may be valuable for evaluating their experiences in ESD-HaH As this was a qualitative design, the aim was not to quantify which of the challenges were most common or had the most influence. Future studies should explore the pervasiveness of these themes through surveys and questionnaires. This study only focused on ESD, and patients might have had different views on the use of HaH in AA. In this study, no caregivers’ perception were studied although they play central role in care of the patients. Most caregivers who are present in the wards are hired formal caregivers who do not have role in decision making. Interviewing informal caregivers (family members) may likely enrich the perspectives of the study.

In conclusion, our findings suggest that patients had positive views toward ESD for HaH and thought it was a viable care model. Addressing concerns and expectations regarding care adequacy, home environment, social support, technology use, and cost considerations must be addressed to support broader uptake. To improve awareness and build confidence in this model of care, we recommend sustained and targeted public education campaigns such as information brochures at patient fronting areas, multilingual digital campaigns, and involving primary care providers and community health workers to build trust. These efforts are critical to achieve wider uptake of HaH services. This would ultimately facilitate its successful implementation in Singapore and similar urban healthcare systems.

## Supplementary Material

Supplemental Material

## Data Availability

The data that support the findings of this study are available from the corresponding author, CYO, upon reasonable request.
