# Supplementary material for: Perceptions and anticipated factors influencing the adoption of early supported discharge through hospital-at-home
Source: Ann Med. 2025 Jul 28;57(1):2537350. doi: 10.1080/07853890.2025.2537350 (PMC12308867; doi:10.1080/07853890.2025.2537350)
Supplement: Supplemental Material [file IANN_A_2537350_SM4288.docx]

**Supplementary Box S1**

**Semi structured interview guide**

Opening statement:

Mobile inpatients at home (MICH) is a new model of care in which inpatients are treated at home.

1. **Exposure**
2. Have you ever heard of MICH/ Home hospitalization?
3. If yes, what do you understand about this model of care?

*If no, the interviewer is to pass a brochure of MICH to the patient and explain.*

1. **Keenness**
2. Based on your current condition, if this is offered to you, would you be keen to be admitted under this program/transfer your care under MICH/Home hospitalization?
3. Why do you think/opined so?

**C) Challenges**

1. What are the challenges you anticipate if you are admitted to your home?

Note*: If participant unable to anticipate ask around use of telehealth monitoring devices, caregiving for ADL, suboptimal home environment (presence of other family members/residential is co-living with relatives, etc), logistics if were to come back for scans, concerns about inferior care, etc.*

1. (Only for patients admitted to the inpatient ward)

Do you think that early supported discharge from the ward through the MICH/hospital-at-home program equals giving up your hospital bed? What do you think of this?

**D) Finance/ Bill size**

7. The bill size of hospital-at-home could be larger if more healthcare provider visits and investigations are needed throughout the admission. Do you expect to pay less or more when you are admitted to home? By how much percentage?

**E) Enabler/ incentivization**

8. Do you think patients should be incentivized to not stay in the hospital if their condition fits the criteria for home hospitalization? What is a reasonable way of incentivizing?

Supplementary Table 1: Criteria to Hospital-at-Home

| General inclusion criteria | General exclusion criteria |
| --- | --- |
| Stable vital signs.  Able to self-care or have caregiver support.  Have internet or data for connectivity for telemonitoring. | Unstable vital signs.  Requiring telemonitoring more than 6-hourly interval.  Suicidal ideation. |
| Eligible conditions (non-exhaustive) | |
| Cellulitis  Dengue  Gastroenteritis  Gout  Heart failure  Hyperglycemia not in crisis  Intra-abdominal abscess  Liver abscess  Lower back pain  Rhabdomyolysis  Pneumonia  Post-bariatric surgery  Post-minimally invasive surgery  Prostatic abscess  Pyelonephritis  Palliative care needs  Urinary tract infections | |

*Condition-specific exclusions:

For cellulitis: able to be managed outpatient with oral antibiotics, suspicion of necrotizing infection needing surgery

For dengue: severe dengue with significant bleeding manifestation, third space loss, or severe organ involvement.

For heart failure: Has a left ventricular assist device, unable to accurately chart urine output at home, requiring oxygen therapy, significant acute renal impairment Cr>50% baseline, K<3.0

For hyperglycemia not in crisis: evidence of diabetes ketoacidosis (DKA), hyperosmolar hyperglycemia syndrome (HHS), needing sliding scale insulin, on insulin pump, brittle diabetes.

For liver abscess: Requiring daily flushing of drains

For pneumonia: Most recent CURB-65 score >3, cavitary lesion on imaging, pulmonary effusion of unknown etiology, requiring oxygen support.

For post minimally invasive surgery and post bariatric surgery: requiring daily review by surgical team, suspicion of surgical complication requiring another surgical intervention

For pyelonephritis: pregnant woman with pyelonephritis, obstructive pyelonephritis, demonstrable pyonephrosis, clinically well and able to be treated with oral antibiotics.

For rhabdomyolysis: creatine kinase less than 40,000. McMahon score> 60
